# Precision medicine in pediatric oncology

**DOI:** 10.1186/s40348-018-0084-3

**Published:** 2018-08-31

**Authors:** Stefan E. G. Burdach, Mike-Andrew Westhoff, Maximilian Felix Steinhauser, Klaus-Michael Debatin

**Affiliations:** 1 Department of Pediatrics and Children’s Cancer Research Center (CCRC), Technische Universität München, Koelner Platz 1, 80804 Munich, Germany; 2 Department of Pediatrics and Adolescent Medicine, Ulm University Medical Center, Eythstr. 24, 89075 Ulm, Germany; 3 CCC München-Comprehensive Cancer Center and German Translational Cancer Research Consortium (DKTK), Partner Site Munich, Munich, Germany

**Keywords:** Childhood cancer, Targeted drug therapies, Immunotherapy, T-cell-based therapy

## Abstract

Outcome in treatment of childhood cancers has improved dramatically since the 1970s. This success was largely achieved by the implementation of cooperative clinical research trial groups that standardized and developed treatment of childhood cancer. Nevertheless, outcome in certain types of malignancies is still unfavorable. Intensification of conventional chemotherapy and radiotherapy improved outcome only marginally at the cost of acute and long-term side effects. Hence, it is necessary to develop targeted therapy strategies.

Here, we review the developments and perspectives in precision medicine in pediatric oncology with a special focus on targeted drug therapies like kinase inhibitors and inducers of apoptosis, the impact of cancer genome sequencing and immunotherapy.

## Introduction

Cancer is a rare disease in children, adolescents, and young adults. In 2015, 2200 patients younger than 18 years were registered in the Federal Republic of Germany (*Deutsches Kinderkrebsregister*), yielding an incidence rate of 173/1000,000 [1]. In the USA, 15,000 cases of cancer in children and adolescents up to 19 years of age were observed in 2014 [2]. With 186/1000,000 the incidence in the USA is slightly higher than in Germany. This finding is to the best of our knowledge both underappreciated and unexplained. Leukemia, lymphomas, myeloproliferative diseases, and myelodysplastic syndromes are, with 45%, the most common malignant diseases in childhood and adolescence, with acute lymphoblastic leukemia (ALL) being by far the most frequent one. Tumors of the central and peripheral nervous system are second, with about 30%, followed by embryonal tumors (blastomas) and sarcomas.

Treatment of children and adolescents with cancer is one of the major success stories of medicine in general and of clinical oncology in particular. The first promising results were achieved in the 1970s, especially in ALL treatment [3, 4]. The starting point of this success was the implementation of cooperative clinical research trial groups comprising all medical centers treating children and adolescents with cancer. Thus, treatment experience of large numbers of patients could be combined, developed, and standardized. The second key to success was the interdisciplinary multimodal therapy approach. In acute leukemia, this was achieved by polychemotherapy protocols, utilizing combinations of different drugs sequentially. These therapy protocols, which were developed in Europe and the USA, particularly in Germany by the BFM study group, played a significant role in achieving present results. Similar therapy protocols were developed, in Germany in particular, for virtually all pediatric solid tumors, some of which are now being used across Europe in international consortia. Overall, patients are being treated in more than 60 trials and registers, e.g., Ewing 2008 for Ewing and CWS SoTiSaR for soft tissue sarcomas, SIOP-LGG for low-grade gliomas, NB 2004 for neuroblastoma, etc. As a result data of roughly 60,000 patients registered at the German Childhood Cancer Registry (Deutsches Kinderkrebsregister, Institut für Medizinische Biometrie, Epidemiologie und Informatik, Mainz) are available for investigations.

**Fig. 1:**
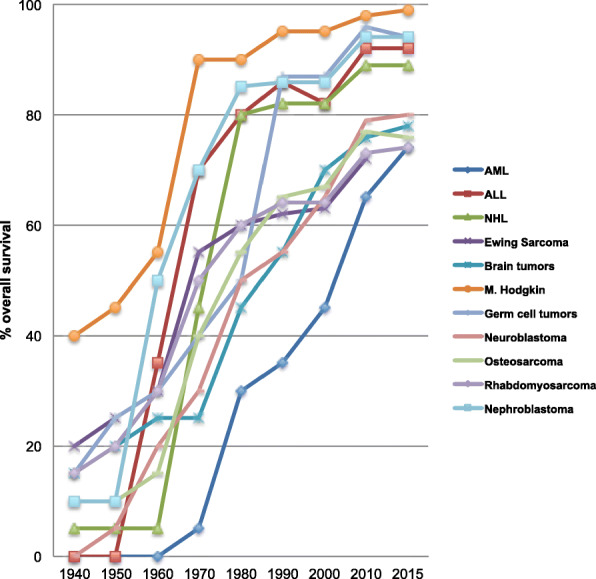
Increase in survival rates in Germany. 2-year-survival until 1980, 5-year-survival from 1980 [1]

Due to both cooperative protocols and multidisciplinary treatment, survival increased dramatically. Up to the early 70s, the 10-year survival rate of patients younger than 20 years was below 20%. Today, 83% of these patients are alive 10 years after initial diagnosis. However, the overall success of pediatric oncology is largely due to excellent outcomes in the treatment of more common cancers [1] (Fig. 1). Successful outcome in ALL improved to over 85% of all cases [4]. In the same period, outcome increased to greater 90% in Hodgkin’s and nearly 90% in non-Hodgkin lymphoma. In contrast, in some cancer entities, particularly in rare tumors, the prognosis is still unfavorable. This applies above all to diffuse pontine glioma and subgroups of other brain tumors, but also some metastatic or refractory sarcoma and blastoma subgroups.

Multimodal therapy in pediatric solid tumors usually still consists of a combination of chemotherapy, radiation, and surgery. A neo-adjuvant chemotherapy approach has been developed for most entities. It provides information about chemotherapy response, while shrinking the tumor, which makes surgery suitable and non-mutilating in numerous cases and may reduce radiation fields in some.

For almost three decades pediatric oncologists tried to cure refractory patients by increasing the intensity of the failing nevertheless already toxic therapy [5]. This was done in the assumption that childhood cancer is a systemic disease in most cases. Even though present imaging methods show tumors still to be localized at initial diagnosis, tumor cells have often already spread. These micrometastases eventually result in overt metastases and relapse. The rationale for intensification of chemotherapy was to reach those metastatic and presumably more resistant cells. In more recent years, however, the long-term side effects—particularly the detrimental effects of radiation and chemotherapy—moved into focus. Moreover, intensification of therapy for most tumor entities, particularly after relapse, improved therapy outcome only marginally. Hence, it is necessary to develop novel and less toxic, e.g., targeted therapy strategies.

Impressive progress in cancer research, including the elucidation of the malignant phenotype and its development as well as the identification of genes causing and driving malignancy, has helped us to define the hallmarks of cancer. These hallmarks describe the deregulation of normal proliferation, migration, vascularization, metabolism, cell death, and survival; and portray the mechanisms of this deregulation as potential therapeutic targets. Those targets are ideally unique features of the malignancy, distinguishing it from its normal counterpart in the tissue of origin [6]. In contrast to conventional chemotherapy, which destroys both tumor and normal cells, precision medicine, by uncovering molecular alteration of the malignancy, aims at targeting cancer cells specifically. Since molecular biology reveals mechanistic differences in conventional entities, it also generates more heterogeneity and makes rare diseases even rarer. As a consequence, cancer is to be treated in a more personalized way [4, 7].

In addition to the advances of targeted attacks on gene products maintaining malignancy, we have experienced breakthroughs in cancer immunotherapy during the last 5 years. In principle, the immune system has cytotoxic mechanisms, capable of killing any kind of altered cell. This is happening permanently, e.g., in case of viral infections by adaptive cellular immunity, and in defense against bacterial infections through antibodies and innate cellular immunity, i.e., phagocytes. The past decade has shown that the immune system, apparently “blinded” by tumor cells during their coevolution in the host, can actually regain its general ability to execute its inherent cytotoxic capacity on tumor cells. Furthermore, the identification of targets on the cell surface made it possible to hit tumor cells with specifically tailored antibodies and hence to pursue new therapeutic options. All these approaches have in common that they target molecularly defined structures and mechanisms with precision tools. Presently, there are more than 40 ongoing clinical trials (www.clinicaltrials.gov) in pediatric oncology worldwide, where innovative therapeutic approaches with new drugs or immunotherapeutics are being tested.

## Review

### Targeted drug therapies

#### Stratification and personalization

Personalized cancer therapy, is not a new concept, particularly in pediatric oncology. Established pediatric protocols stratify patients into different risk groups according to therapy response, i.e., prednisone response and residual disease in acute lymphoblastic leukemia (ALL). Even in the era before response stratification, patients were stratified according to age and gender, leukemic load, and biomarkers, e.g., a T-cell ALL occurring on teenage boys was stratified into a higher risk group compared to common B-progenitor derived ALL. T-lineage persists to date as an initial biomarker of risk of relapse.

Furthermore, progress in cytogenetic techniques, and increasingly in genomic analysis, determined that certain risk groups in ALL, defined for instance by chromosomal translocation or by poor in vivo response to therapy (prednisone response on day 8, monitoring of minimal residual disease with molecular markers), are associated with a worse response to therapy.

As a result, more intensive therapies, e.g., allogeneic stem cell transplantation was used in the treatment of t(9;22) positive ALL. Similar risk stratifications are used in nearly every therapeutic trial in pediatric oncology, taking into account tumor localization, extent of spread, histologic subtype and, in recent years, increasingly molecular characterization. Detection of circulating tumor cells and circulating tumor DNA (“liquid biopsies”) plays an emerging role as non-invasive diagnostic and disease monitoring tool. Liquid biopsies are now being evaluated for neuroblastoma and Ewing sarcoma [8, 9].

First in 1960, cytogenetics became crucial for the molecular characterization of cancer [10]. Very early on, karyotyping showed a difference in the number and structure of chromosomes in cancer cells compared to normal cells [11–14]. This resulted in the characterization of translocations that have been found in leukemia as well as in solid tumors such as Ewing and other sarcomas. Moreover quantitative genomic alterations can be characteristic of certain cancers like the amplification of the super-enhancer MYCN (myelo**c**ytomatosis oncogene of neuroblastoma), a member of the MYC superfamily and of the 450 Ma old MYC interactom [15], driving most notably high risk neuroblastomas. Discovery and exploration of oncogenes have decisively influenced therapeutic concepts and laid the foundation for today’s quantum leap in knowledge and possible therapies.

#### Precision medicine and targeted therapies

Over the past two decades, basic research expanded our understanding of fundamental aspects of cancer. Malignant deregulation of cells is due to genetic changes in somatic cells through mutation, translocation, or overexpression of genes, resulting in cellular dedifferentiation, proliferation, avoidance of cell death, and survival under cellular stress. This knowledge rendered altered genes or their genetic products, proteins, and targets for therapeutic interventions. The prime example, delivering a breakthrough in therapy, was the analysis of the Philadelphia chromosome. The translocation t(9;22) results in the BCR-ABL (breakpoint cluster region—Abelson murine leukemia viral oncogene homolog 1) fusion gene. It activates the tyrosine kinase ABL, causing autonomic proliferation in affected cells. This genetic alteration exists not only in chronic myeloid leukemia, but also in childhood acute lymphoblastic leukemia, albeit in a low percentage of patients [16].

Through the application of a tyrosine kinase inhibitor, imatinib, the induction of proliferation through ABL-activation can virtually be stopped completely and leukemic cells die. Treatments with imatinib, for example, in chronic myeloic leukemia, as monotherapy can lead to long lasting molecular remission and operational cure [17]. In ABL-activated acute leukemias, however, imatinib is only beneficial when incorporated into combined cytotoxic regimes.

In pediatric oncology, as in adult medicine, many current molecular profiling programs for patients with relapsed or refractory tumors aim to sequence tumor genomes, in order to identify genetic alterations and switch off related genes with targeted therapies. In Germany, the Society for Pediatric Oncology and Hematology (GPOH) is promoting the INFORM trial. Sequencing is performed centralized at German Cancer Research Center (DKFZ) while most of the German pediatric oncologic centers take part [18]. In the USA, the National Institute of Health and the Children’s Oncology group are performing the Pediatric MATCH trial (Molecular Analysis for Therapy Choice) [19–21]. Presently, the results of at least eight such trials have been published or presented at symposia [19, 21–27]. Depending on the trial, between 50 and roughly 300 samples from patients with primary disease, relapse, or refractory disease were sequenced per study. Overall, a genetic alteration that can be targeted therapeutically, i.e., for which drugs already exists, could be identified in between 10 and 30% of patients [18, 19]. These results are sobering and might even be over-optimistic, since it very much depends on the definition of targetable alterations, differentiation between driver and passenger mutations, detection of the frequency of gene fusions or minor clones, drug interactions, pharmacology, and last not but not least bioinformatics [28]. Even in the presence of a targetable alteration, available drugs may substantially differ in their efficacy depending on the cellular context. We have learned, for instance, that neuroblastoma bearing activating anaplastic lymphoma kinase (ALK) mutations respond much less efficiently upon crizotinib treatment than ALK translocations in lymphoma or lung cancer [29]. Beyond that, some trials in adult medicine that have been published did not reveal an advantage over “physician’s choice,” even, when targetable genetic alterations were identified and appropriate drugs administered [4]. Finally, as long as targeted therapies do not target oncogene addiction pathways, they may well prime for resistance, raising selective pressure to bypass the targeted pathway with alternate rescue signaling [30].

Moreover the new paradigm of dichotomy between proliferation and metastasis [31] deserves consideration in this context. Downregulation of pathognomonic fusion proteins such as EWS/ETS may be associated with reduced tumor proliferation but increase of migration and consecutive metastasis: in Ewing sarcoma (ES) the level of fusion gene expression correlates inversely with a tendency toward metastatic spread [32]. We [33, 34] as well as researchers in the USA [35] have further shown that gene products, which are overexpressed in ES, may inhibit growth of the primary tumor but favor metastatic spread [36].

#### Somatic vs. germ-line genetic alterations

It is widely accepted that, in contrast to adult cancers, most childhood cancers develop as an accident of growth or differentiation and not as a result of environmental mutagenic impact. It has also been assumed that, apart from rare hereditary cancer syndromes, there is no genetic predisposition. The trials cited above have now opened a new view on malignant diseases in children, adolescents, and young adults [37]. It has been shown in six independent studies that 5–10% of patients have germ-line mutations that predispose to cancer [18, 19, 21, 24, 25, 37, 38]. This is all the more surprising because these mutations affect primarily patients from families where there is no increased susceptibility for cancer, i.e., high incidence of malignant diseases or cancer in adolescence. Thus, we have to assume that they are de novo mutations and yet to understand many consequences of these findings, particularly for targeted therapies.

Another take home message from these trials is the significant difference in the number of mutations between cancer in childhood and adolescence vs. cancer in adults. Whereas virtually all cancers in elderly patients have multiple genomic alterations, where tumor cells are often polyploid with multiple aberrations of chromosomes; most pediatric tumors however, exhibit only few mutations and genetic alterations [4, 18]. This limits the availability and use of drugs for targeted therapies. One has to take into account, however, that most of the trials carried out so far focused on identifying mutations. In a lot of cancers the aberrant expression, overexpression and deregulated activation of certain genes is causative. Genomic rearrangements in non-coding regions may lead to massive activation of oncogenes, such as GFI1 (growth factor independent 1 transcriptional factor) in medulloblastoma or TERT (telomerase reverse transcriptase) in neuroblastoma [39, 40]. We are still missing systematic functional trials addressing this issue. Only a single institution transcriptomic trial from one of the authors’ institution revealed druggable targets in all patients and a survival advantage of patients with targeted therapies [22]. Nevertheless, together these findings highlight that expression of targets on the protein level need to be verified as well as delivery of targeting drug. As an exception to the general rule of low mutational load in childhood malignancy vs. high mutational load in cancer in the old, the hypermutational load of the malignancies caused by germ line encoded mismatch repair deficiency syndromes, has provided a successful rationale for T-cell checkpoint inhibition and for the avoidance of genotoxic therapy in these young patients [41]. Thus, more experience is to be gained, and more complexities have to be understood, before precision medicine can realize its innovative potential and strengths.

#### Precision medicine: tyrosine kinase inhibitors, inducers of apoptosis, and other cell modulators

The terms “precision medicine,” “personalized medicine,” and “individualized medicine” are now part of medical concepts that seek to identify and target molecular structures in many diseases, including cancer. Former US President Barack Obama launched the Precision Medicine Initiative in 2015 (“Cancer Moon Shot Initiative”), comparing it to the first moon landing. The US National Institutes of Health are using this initiative to form new strategies for diagnosis and therapy, particularly of cancer [7].

**Fig. 2:**
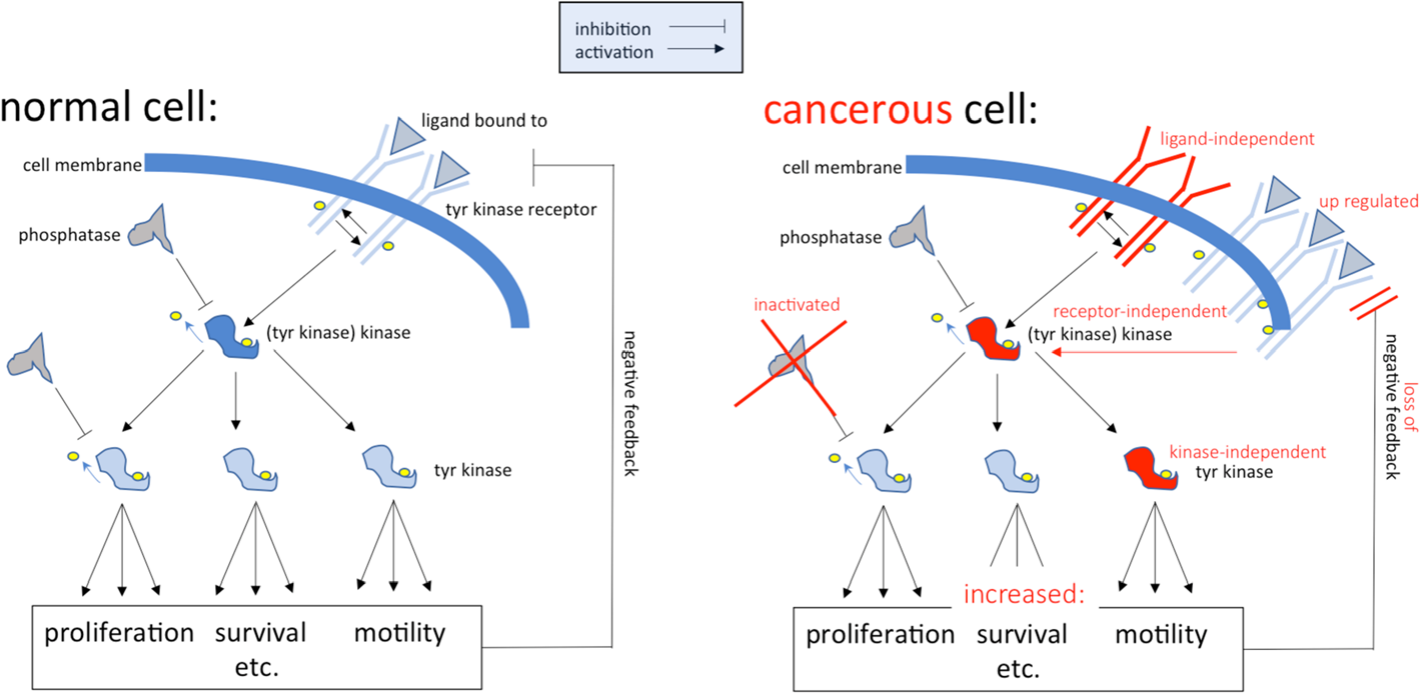
Aberrant activation of tyrosine kinases as a mechanism for malignant transformation. Cancer cells are defined by overactive signaling cascades, often mediated by tyrosine (tyr) kinases. Common therapeutic strategies are either blocking of the tyr kinase receptor by inhibiting antibody/pharmacological inhibitor (which does not work for ligand-independent signals and has reduced potency if the target is overexpressed), or utilizing pharmacological inhibitors that block kinase activity (dependent/independent of mutational status) [42]

A hallmark of cancer is the activation of genes forcing cells into proliferation or maintaining survival under stress while blocking differentiation and cell death [6]. Normally, cells receive external signals that are transmitted into the cell by receptors, for instance tyrosine kinase receptors transmitting signals through phosphorylating tyrosine residues of proteins. Deregulated activation of tyrosine kinases is characteristic of most cancers [42] (Fig. 2).

However, identification of the specific kinase in each case may not be easy, as deregulated activation can arise not only from activating mutations but also from inactivating mutations in suppressors (Table 1). The identification of tyrosine kinase inhibitors, initially in adult cancers, has provided a spectrum of substances that can be used therapeutically, as shown with imatinib in Philadelphia chromosome positive ALL and CML. In most cases, however, these drugs are effective only for a short time (with the exception of CML) when used in in monotherapy (cf. 2.2.) [30].

**Table 1:** Kinase-inhibitors and other targeted agents in pediatric malignancies

Genomic alteration	Target structure	Medication	Example pediatric tumor
ALK mutation/fusion	ALK	Crizotinib	Neuroblastoma
			Embryonal tumors
MYCN amplification	AURKA	Alisertib	Neuroblastoma
BRAF mutations/fusions	BRAF	Vemurafenib	Melanoma
		Dabrafenib	Langerhans-cell histiocytosis
			glioma
FGFR1/2/3 fusion, amplification, mutation	FGFR	Dovitinib	Rhabdomyosarcoma
		Ponatinib	Ewing Sarcoma
N/KRAS mutation	MEK	Trametinib	Melanoma
PTPN11 mutation		Selumetinib	Glioblastoma
			Juvenile myelomonocytic leukemia
BRCA1/2 mutations	PARP1	Olaparib	Osteosarcoma
EWSR1-FLI fusion		Rucaparib	Ewing sarcoma
ATM mutation			
loss of PTEN	PI3K/mTOR	Everolimus	Sarcoma
PIK3CA mutations		Temsirolimus	
		Rapamycin	
PTCH1 mutation	SMO	Vismodegib	Medulloblastoma
FLT3 mutation	Multikinases	Sorafenib	Acute myeloid leukemia
or internal tandem duplication			
VEGF Receptor	Multikinases	Pazopanib,	Sarcoma
Expression of cKit and PDGF receptor		Regorafinib	Ewing sarcoma

There are interesting examples of genetic alterations that were initially identified in different adult cancers and are now being targeted in pediatric oncology. The most prominent example is ALK, which was initially identified in anaplastic large cell lymphoma and later found in a significant share of neuroblastoma patients and in high frequency in lung cancer with activating mutation [43, 44]. Although the specific kinase inhibitor crizotinib was not very effective in the treatment of patients with high-risk neuroblastoma or relapse, newly developed ALK inhibitors such as ceritinib or lorlatinib may be more effective in this malignancy and are now being evaluated in clinical trials [29, 45, 46].

Analysis of gene-expression profiles can lead to the identification of patterns, which can then be targeted with different tyrosine kinases. Philadelphia-like ALL is exemplary here. The driver translocation t(9;22) of Philadelphia (Ph) ALL, results in the overexpression of the ABL oncogene, activating other tyrosine kinases itself. This spectrum of activation of different tyrosine kinases can also be found in samples of ALL missing the Ph translocation. This Philadelphia-like leukemia has an unfavorable prognosis, similar to Ph positive ALL, and can at least be co-treated successfully with tyrosine kinases as well [47].

There is a broad spectrum of diseases in pediatric oncology, where tyrosine kinase inhibitors can be used. Lesions thereof are being found in a minority of patients with a variety of cancers. Therefore, their use is only feasible and appropriate in the context of trials after sequencing has been performed. This concept of therapeutic personalization has lead to novel designs of clinical studies such as basket (same target in different entities) and umbrella (different targets in same entities) studies. Selected examples are shown in Table 1.

Apart from tyrosine kinase inhibitors, there is interest in other therapeutic strategies that aim to influence cell survival in general or target the “motor” independent of possible mutations [48–51]. Amongst those are strategies that target proteins of the BCL (B-cell lymphoma)-2 family, which inhibits programmed cell death. Preclinical data suggests that high-risk patients in ALL or neuroblastoma could benefit from treatment with BCL-2 inhibitors [52, 53].

Here, too, findings in adult cancers were pioneering. Chronic lymphatic leukemia, defined by differentiation of B-lymphocytes and virtually untreatable through chemotherapy, shows an excellent and long-lasting response to the BCL-2 inhibitor venetoclax, which has recently been approved for clinical use [54].

Detailed genomic analysis has led to new definitions of different tumor entities, which were previously thought to be uniform. In medulloblastoma, there are four clearly distinguishable, molecularly defined subgroups with different genetic alterations that result in deregulated signal transduction, i.e., WNT (wingless), SHH (sonic hedgehog) (both named for altered signal transduction pathways), group, 3 and group 4. These subgroups are prognostically relevant, with WNT having the best and group 3 having the worst prognosis. Their molecular profiles can provide possible targets for approaches in precision medicine. Genome sequencing in the SHH subgroup, e.g., can predict whether a tumor is responsive to inhibition of the Smoothened (SMO) protein [55, 56].

#### Perspectives of targeted therapies

The analysis of tumor genomes led to substantial insights into cancer development. Genomic analyses can provide biomarkers and identify novel targets for targeted therapies. Nevertheless, there are limitations: while there are bona fide examples for significant improvement of survival due to implementation of targeted therapies in adult cancers (such as treatment of EGFR-mutated lung cancer), not all hopes have been fulfilled due to primary or secondary resistance [57]. Furthermore, there is an intrinsic problem within the tumor itself. In many cases, tumors show extensive genetic heterogeneity, e.g., structural heterogeneity in osteosarcoma due to mutations in DNA repair [58]. Genetic heterogeneity can be found in solid tumors as well as in leukemia. It implies that different cells have different genetic alterations. It thus can be assumed that several clones exist at diagnosis. Relapse can arise by evolution from a preexistent subdominant clone resistant to therapy; these cells may not be detectable initially [59–61]. Targeted therapy may thus have to aim at moving targets. This suggests a combination of therapies addressing different structures and signaling pathways. These results also suggest that the ability of the immune system to control and eliminate tumor cells has to be employed more often, if necessary, in combination.

In summary, the molecular analysis of tumors and leukemia in childhood and adolescence has made groundbreaking progress in our understanding of cancer. Furthermore, possible targets for specific therapies in the context of precision medicine have been identified. These therapeutic approaches may prove their efficacy in clinical trials and reduce the grave side effects of conventional cytotoxic therapies.

### Immunotherapy

#### Evolution and function of the immune system

How long does it take from a scientific breakthrough in basic research to clinical application? 20, 50, or 100 years? All answers are correct for immunotherapy of cancer depending on which groundbreaking discovery you want to take into account [62–71]. History reveals that translational research may reduce the latency period. Even in December 2013, when Science magazine picked cancer immunotherapy as the breakthrough of the year, there were still serious doubts amongst the jurors about whether this breakthrough would lead to a sustainable change in clinical practice. Today, immunotherapy is becoming the fifth modality in cancer therapy (next to surgery, radiation, chemotherapy, and targeted therapy).

The adaptive (or specific) immune system has two evolutionary related effector mechanisms: humoral and cellular immunity. Antibodies are the effectors of humoral immunity. They are the older extant within the evolution of the adaptive immune system. Antibodies are produced by B-lymphocytes and bind to molecules on the surface of target cells; thus, the repertoire of antibodies is limited to those target molecules that occur on the outer cell membrane of blood cells or cellular organisms circulating in the blood, i.e., bacteria. Effectors of humoral cytotoxicity are myeloid cells of the innate immune system (i.e., phagocytes, antibody dependent cellular cytotoxicity, ADCC) or the complement system (complement dependent cytotoxicity, CDC). Antibodies developed earlier than jawed vertebrates in evolution and the inborn immune system (innate, non-specific or natural immunity) is evolutionarily older than the adaptive immune system. It can already be found in plants.

**Fig. 3:**
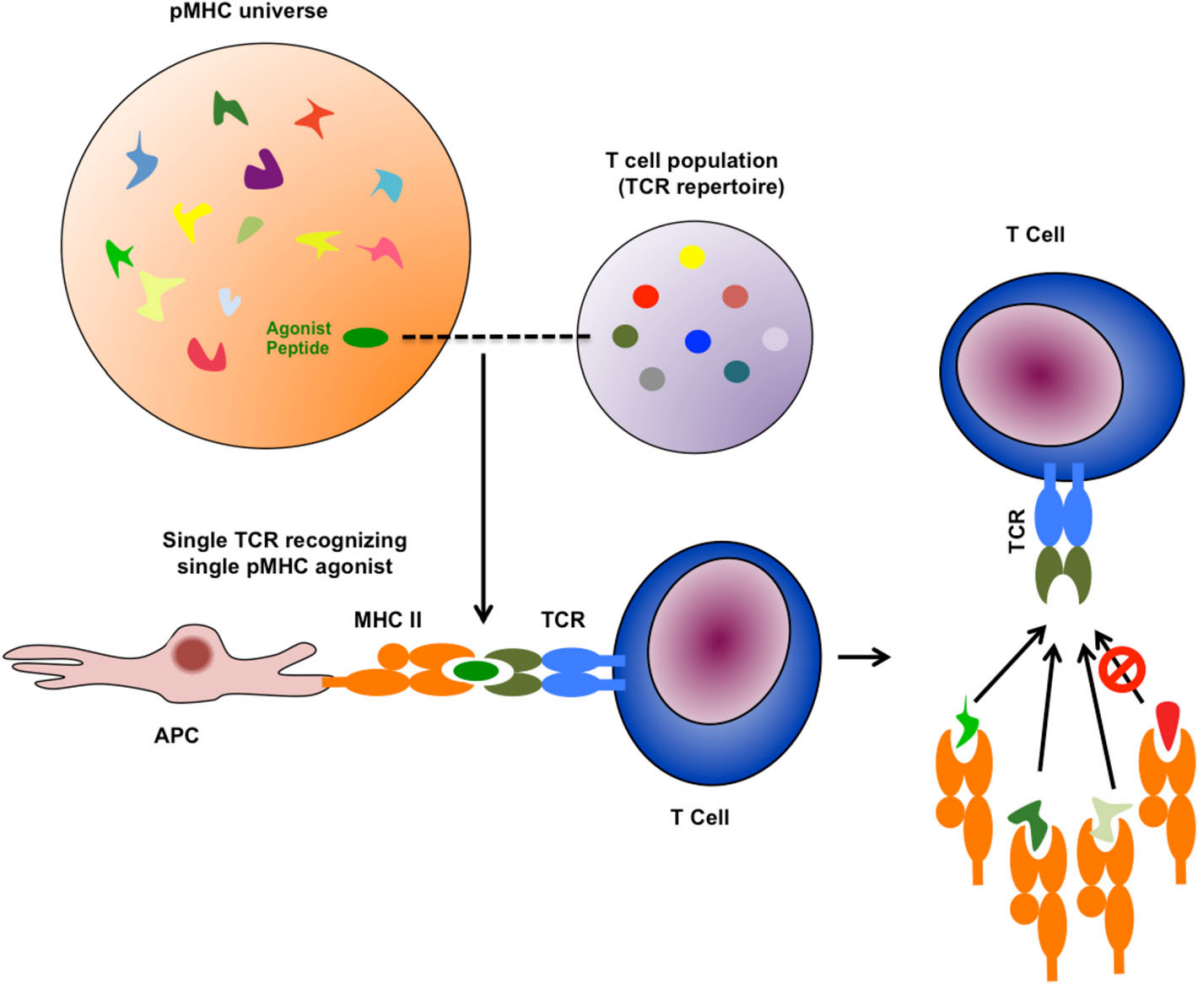
10^11^ human TCRs have to recognize 10^20^ peptides: a TCR repertoire (purple sphere) is several magnitudes less diverse than the total set of peptides that are presented by MHC molecules (pMHC) (orange sphere). Hence, a necessary feature of a TCR repertoire is that a T-cell is able to recognize and respond to many peptides, but one TCR only recognizes and responds to peptides closely related to the original agonist peptide (similar colors representing peptide relatedness). Modified from Mandl and Germain 2014 [73]

Apart from their special teeth, carnivores were the first jawed vertebrates (gnathostomata) to develop a cellular adaptive immune system to reject the cells of their prey including incorporated pathogens and defend themselves against hostile takeover by their victims [72]. Effectors of cellular adaptive immunity system are T-lymphocytes. Through T-cell receptors (TCRs), they recognize endogenous and exogenous protein fragments (peptides) being presented by the major histocompatibility complex (MHC, in humans: human leukocyte antigens, HLA). In contrast to bacteria, viruses require host cells for replication and thus are primarily controlled by T-cells in contrast to B cells primarily controlling bacteria. However, the evolution of immunity, i.e., immunologic memory, requires interaction between the innate and adaptive immune system as well as B- and T-cells in the latter. Paradigmatically, every universal peptide can be recognized by the cellular adaptive immune system. Thus, the TCR-repertoire has been termed unlimited. However, the relation between the possibility of recombination of the TCR and the number of universally possible peptides implies an imperative TCR promiscuity: 10^11^ human TCRs have to match with 10^20^ peptides [73] (Fig. 3).

To execute the cytotoxic function, T-cells perforate the cellular membrane of the target cell with perforin and instill the cytotoxic protein granzyme B into the cytosol of the target cell.

#### Therapeutic modalities utilizing innate immunity

More than 100 years ago (clearly before the advent of radio- and chemotherapy), the American surgeon William Coley treated sarcomas, amongst them Ewing sarcomas, successfully by inoculation with Coley’s toxin, a mixture of attenuated *streptococci* and *serratiae*. This unspecific immunotherapy is based on a stimulation of the inborn immunity resulting in an inflammatory reaction that can elicit an anti-tumor effect.

The high frequency of GC in mycobacterial DNA acts as a signaling pattern eliciting an innate immune response with consecutive T-cell stimulation. About 50 years ago, Mathé developed the inoculation with bacille Calmette-Guérin (BCG), which is still approved today in the treatment of bladder cancer. A newer innovation based on the immune stimulatory effect of mycobacteria is the macrophage-activating drug mifamurtide (muramyltripeptide, Mepact®). Mifamurtide is a synthetic analog of muramyldipeptide, an immunogenic component of the mycobacterial pathway. The immune stimulatory effect of mifamurtide is mediated via the binding of NOD2 (nucleotide-binding oligomerization domain protein)-receptors on monocytes, macrophages, and dendritic cells. Mifamurtide was approved in the EU in 2009, about 30 years after it was developed, as an orphan drug for the treatment of osteosarcoma. Apart from myeloid-derived monocytes and macrophages, lymphoid-derived natural killer cells (NK) are the cellular effectors of innate immunity. NK cells can elicit antitumor effects, especially in acute myeloid leukemia and solid tumors in adults.

#### Therapeutic modalities utilizing adaptive immunity

##### Vaccination

Vaccination induces a memorized response of the cellular and humoral adaptive immune system. The peptide vaccination trial in children with relapsed acute lymphoblastic leukemia (iVac-ALL) is a well-developed approach of this immunotherapeutic strategy [74]. Nevertheless, it addressed only random mutations, whose relevance for the persistence of leukemia is unknown. Results for several vaccination trials against pediatric solid tumors have been published, amongst them high and low grade glioma [75, 76], atypical teratoid-rhabdoid tumor [77], hepatoblastoma [78], and neuroblastoma [79], showing antigen-specific immune response and even improved survival in high-risk sarcoma [80]. Most recently, it has been shown that STING cytosolic DNA sensing may have also play a role in vaccination [81].

##### Monoclonal antibodies

*Rituximab* is a CD20 antibody, used in the treatment of malignant lymphoma (EU-approval in 1998) and the first antibody to be approved for the treatment of cancer (FDA-approval in 1997). Rituximab is also indicated for the treatment of post-transplant lymphoproliferative disease (PTLD) [82]. Furthermore, it is used in the treatment of autoimmune diseases such as rheumatoid arthritis, idiopathic thrombocytopenic purpura (ITP), and lupus-associated nephritis [83].

*Dinutuximab* beta (APN311, ch14.18/CHO) is a chimeric monoclonal antibody recognizing specifically the glycolipid GD2, a membrane-bound molecule, expressed in high-frequency on neuroblastoma cells. ADCC and CDC mediate the antitumor effect of the anti-GD2 antibody [84].

While rituximab and dinutuximab elicit their antitumor effects via the natural effectors of humoral cytotoxicity ADCC and CDC, the anti-CD30 antibody *brentuximab* is a conjugate of an antibody and a cytotoxic agent; these conjugates contain, e.g., cytostatics as cytotoxic pay load. Brentuximab is approved for the treatment of adult Hodgkin’s disease and anaplastic large cell lymphoma (ALCL).

*BiTE antibodies* (bi-specific T-cell engagers) are bi-specific monoclonal antibodies. They consist of two single chain variable fragments (scFV), connected via a peptide bridge. BiTE-antibodies can thus specifically recruit T-cells to tumor cells to execute a T-cell-mediated immune response. Blinatumomab is the first clinical grade BiTE-antibody. It recognizes CD19 as well as CD3 (which is expressed on T-cells) and brings T-cells into direct contact with B-cell ALL, so it can be eliminated by cytotoxic T-cells [85]. Blinatumomab is now being approved in adults and used off-label in children with relapse of B-cell ALL (NCT02101853). BiTE-anitbodies use the same mechanism as chimeric antigen receptor (CAR) transgenic T-cells.

##### T-cells: DLIs, TILs, and checkpoint inhibitors

In 1986 Rosenberg at the NCI demonstrated that interleukin 2 activated T-cells (tumor infiltrating T-cells, TILs) infiltrate and at least temporarily eliminate tumors. In 1990, Kolb demonstrated in Munich that donor lymphocyte infusions (DLIs) induce remission in chronic myeloid leukemia. DLIs are also effective in several pediatric neoplasias like AML [86] and advanced pediatric sarcomas [87]. Allison showed in 1996 for the first time, that blocking inhibitory receptors on tumor-infiltrating T-cells can be therapeutically effective. The antibodies he developed against those inhibitory receptors have become a new class of substances in cancer therapy known as checkpoint inhibitors. They play an emerging role in the treatment of adult cancers, for example, Hodgkin and non-Hodgkin lymphoma [88]. With present protocol designs, however, they have not shown to be effective in most childhood cancers, except mismatch repair deficiencies [41] (cf. 2.3.). In contrast to the latter, most childhood cancers have a low mutational burden and are thus thought to be poorly immunogenic.

Meanwhile, the Rosenberg group and its spin-offs expanded their approach by screening whole-exome-sequencing data to identify mutant proteins. They synthesized mutant epitopes of TCR recognition that had been established by a major histocompatibility complex-binding algorithm for TILs. With this approach, they identified mutant antigens expressed on autologous tumor cells and recognized by TIL lines of melanoma patients, who experienced tumor regression after adoptive T-cell transfer. This is a straight method to identify mutant antigens that are recognized by T-cells. The methodology could evolve as a blueprint for a general approach for the identification of mutant antigens expressed by different tumor types [86]. Due to the generally low mutational load of childhood cancers, its relevance here may be restricted, e.g., to DNA repair deficiency syndromes.

##### CAR T-cells

The most important breakthrough in cellular immunotherapy for pediatric oncology was the development of chimeric antigen receptor (CARs) transgenic T-cells targeting CD19. Antibodies bind membrane-bound molecules on target cells with high affinity. T-cells have a potent cytotoxic machinery but a low binding affinity as well as a MHC restriction of target structures. The separation between antibody binding and cytotoxicity is an evolutionary safety mechanism that is circumvented by CARs. This technology was introduced in 1993 when Eshhar et al. a conjugated an immunoglobulin V-region with a T-cell activating molecule by transfection into cytotoxic T-cells [89].

CD19 is an antigen on the cell surface, which can be found on most B-cell derived ALLs. Many teams developed and optimized strategies to transduce autologous T-cells with CD19 antibody fragments that are connected to various intracellular domains of the T-cell receptor. These T-lymphocytes can thereby recognize CD19 on B-cell ALL cells and eliminate them. They are termed chimeric antigen receptor T-cells since the antigen binding part of the T-cell receptor is functionally replaced by a membrane-bound antibody. CAR T-cells are a novel therapeutic option, which has been approved by regulatory authorities in the USA.

**Fig. 4:**
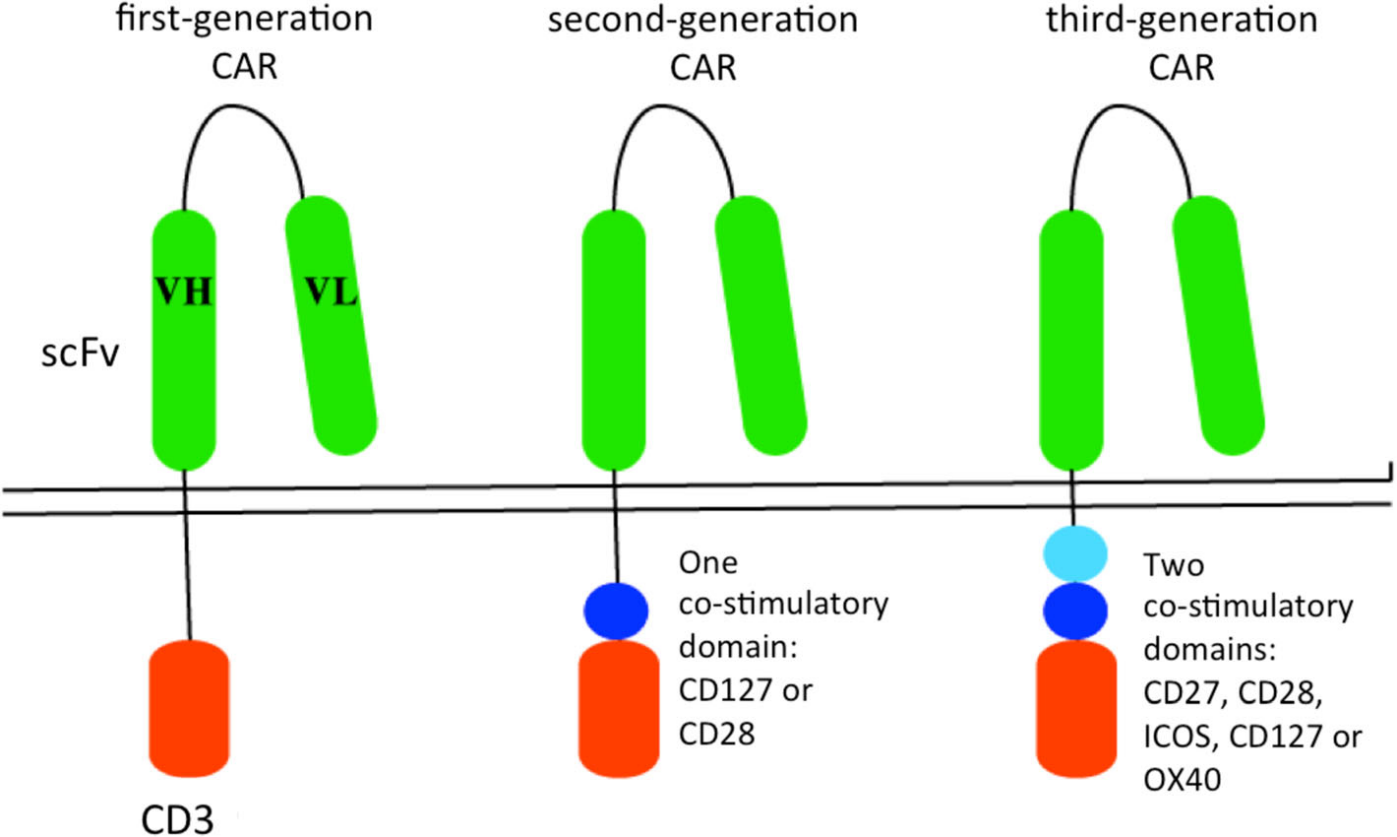
Immunoglobulin/T-cell receptor constructs for the generation of CAR T-cells [91]

**Fig. 5:**
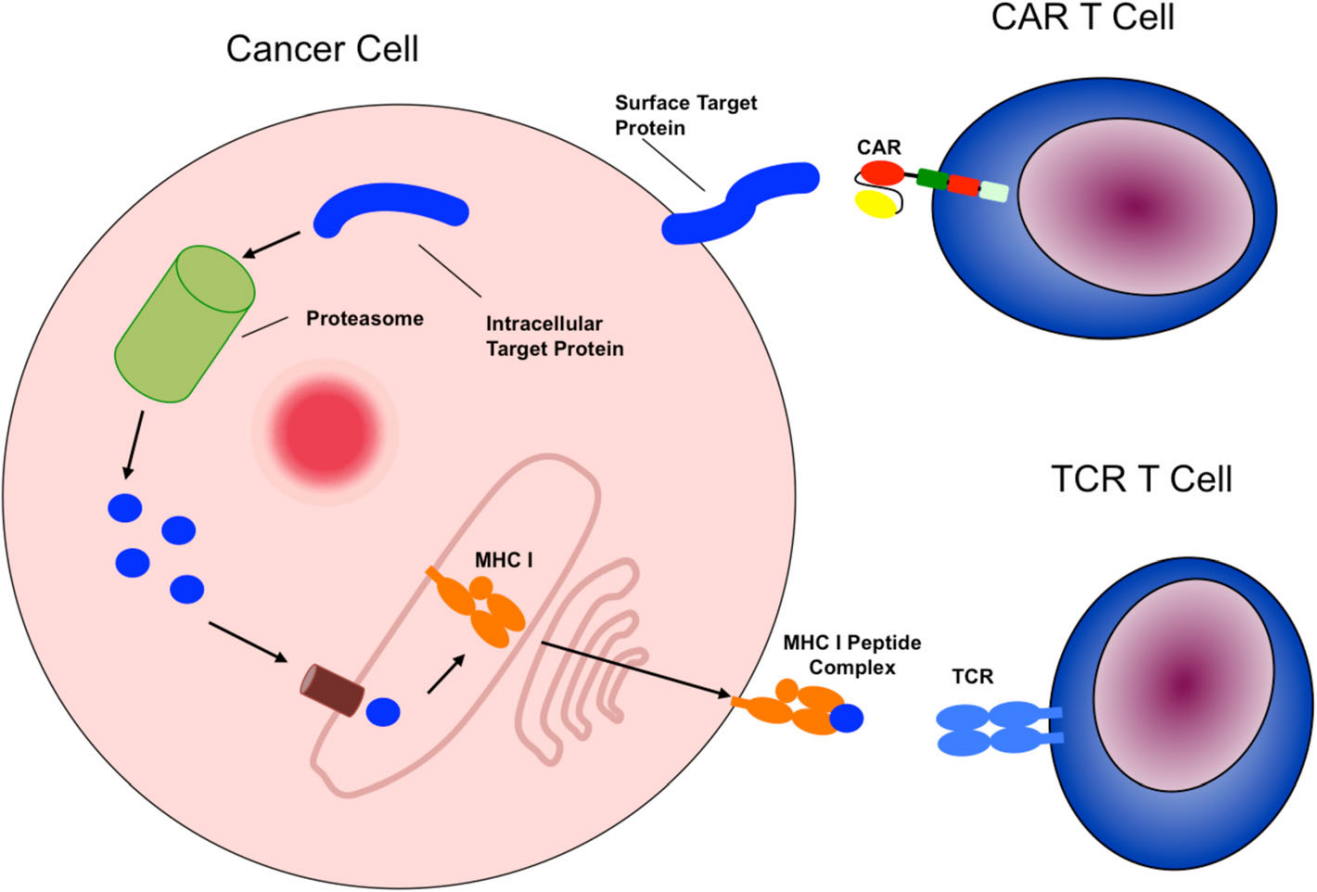
Cytotoxic mechanisms by TCR T-cells and CAR T-cells

Claudia Rössig and Malcolm Brenner published results on CAR T-cells 15 years ago. However, due to regulatory sponsorship challenges involving the European Society for Blood and Marrow Transplantation (EBMT), their long planned European trial for the treatment of ALL with CD19 CAR T-cells of the first generation could not be realized [90]. Of note, these first generation CAR T-cells were safer but less efficacious, since they did not contain the costimulatory domains of later generations. Meanwhile Carl June developed second-generation CAR T-cells (Fig. 4 [91]) at the Children’s Hospital of Philadelphia, they had, meanwhile, been approved by the FDA [92]. In a phase 2, single-cohort, 25-center, global study 75 patients suffering from refractory or relapsed ALL with have been treated with these CAR T-cells: the overall remission rate within 3 months was 81% ongoing remission in 60% between 8 and 18 months. Event free survival is 50% 12 to 20 months after the infusion. Forty-six percent suffered from severe (grades 3 and 4) cytokine release syndrome (CRS) and 5% from grade 3 encephalopathy after T-cell activation in vivo. Total incidences of CRS and neurologic events were 77 and 40% [93]. Because CD19 is not essential for leukemic cell survival, cells can become CD19-negative due to selective pressure; CD19-negative relapses arise with longer observation times. Their limitation to recognition of structures on the cell surface is a fundamental disadvantage of CAR T-cells (Fig. 5).

Loss of antigen is a central obstacle in cancer immunotherapy. Therefore, efficacious immunotherapy should address targets that are essential for tumor cell survival and metastasis. Attempts to transfer recent achievements of cellular therapies, e.g., CAR T-cells, to the treatment of solid tumors, particularly pediatric sarcoma, have yielded limited success so far. Because oncologic driver genes are often not coding for antigens, further target proteins have to be identified, which are selectively overexpressed and essential for malignancy and metastases. Those antigens can be addressed by TCR driven T-cells.

##### TCR-based T-cell therapy

TCR-based T-cell therapies can, because of their broad repertoire, address molecules that are essential for tumor cell survival (addiction oncogenes) and metastasis. Identification of these proteins and verification of their indispensable function in vivo, presentation of the peptide, and assessment of selective TCR reactivity is essential. Conventional TCR reactivity (i.e., recognizing self or autologous targets) is low against non-mutant peptides of the tumor due to thymic education achieving negative selection of TCRs with high affinity for peptides presented by self MHC. One approach to increase TCR reactivity is improvement of affinity by random mutagenesis. However, affinity enhanced TCR mutants are prone to cross-reactivity with unforeseeable and fatal side effects.

T-cells that do not undergo negative but only positive thymic selection include T-cells of the alloreactive repertoire. Therefore, they are attractive candidates for cancer immunotherapy. Alloreactive T-cells are thought to have a higher risk of cross reactivity compared to conventional T-cells. But these conventional T-cells usually do not eliminate tumors presenting self-peptides with few mutations. This is because T-cells with MHC cross reactivity are the prime target of negative thymic selection.

Therefore, for effective immunotherapy, it is desirable to target proteins essential for tumor cell survival. Chondromodulin-I (CHM1) is a downstream target of the driver oncogene EWS-FLI1 in Ewing sarcoma and promotes metastasis [94].

The use of allorepertoire derived TCRs, which exploit the mechanism of defense against non-self, could improve affinity and overcome anergy of T-cells to self-tumor antigens. At the TUM Children’s Hospital Medical Center München-Schwabing, patients with advanced ES have been treated successfully without side effects utilizing alloreactive TCR transgenic T-cells which targeting ES associated peptides CHM1 and enhancer of zeste homolog 2 (EZH2) presented by non-self MHC [95].

Targeting peptides derived from mediators of metastasis, presented by non-self MHC may solve two fundamental problems of cancer immunotherapy: (1) it renders non-immunogenic tumors susceptible to adoptive and TCR-based therapy, (2) it circumvents immune evasion by targeting mechanisms obligatory for metastasis.

#### Perspectives of immunotherapy

Table 2 gives an overview of progress in precision medicine in immuno- and cell-based therapy in pediatric oncology.

**Table 2:** Immuno- and cell-based therapy in pediatric oncology

Disease	Drug	Target
1. Innate immunity		
1.1 osteosarcoma	Mifamurtide	NOD2
2. Humoral immunity		
2.1 Neuroblastoma	Dinutuximab	GD2
2.2 NHL and PTLD	Rituximab	CD20
2.3 HL and ALCL	Brentuximab	CD30
3. Cellular immunity		
3.1 AML	DLI	Multiple
3.2 ALL	CAR T-cells	CD19
3.3 Ewing sarcoma	TCR T-cells	CHM1

The centennial success of pediatric oncology was based on the multidisciplinary approach involving less mutilating surgery in a neo-adjuvant setting as well the cytotoxic modalities of mutagenic cell toxins (e.g., war-agent derivatives) and ionizing radiation with ensuing long-term toxicity in cancer survivors. Disruptive high-throughput technologies may provide an urgently needed paradigm shift here. The current concept of randomized trials may not fully appreciate the heterogeneity of increasingly subdivided entities and is being replaced by individualized therapies according to genomic and other high throughput analyses. This individualization implies the risk that the efficacy (and even more superiority) of these novel therapies may be difficult to prove when only a few patients are treated with a specific treatment regimen. These challenges have to be addressed by novel study concepts, including adaptive design, basket and umbrella trials, establishing surrogate endpoints (e.g., biomarkers) as well as multi-modal high throughput molecular analyses of individual patients. This individualization of therapy will put the individual patient into the focus of research.

The imminent paradigm shift is based on the assumption that individualized molecular analysis will provide biomarkers for targeted therapies that eliminate malignant but spare normal cells. This assumption has not been proven yet for tumor stem cells. An alternative assumption implies that development of cancer in children is not characterized by an accumulation of oncogenic events, but by a selection advantage of any genetic event favoring dedifferentiation and the reversion to the embryonic default mode. Along with this assumption, it may not be the genetic alterations that are carcinogenic, but a tumor-microenvironment may provide epigenetic and metabolic reprogramming [96]. Reprograming T-cells with chimeric receptors to manipulate selectively that tumor microenvironment may open here new horizons for cancer immunotherapy [97].

### Side effects and draw backs of targeted therapies

While the tools of precision medicine theoretically induce much less systemic toxicity due to their targeted approach than the non-specific cytotoxicity of classical chemotherapy, we had to learn with our ongoing clinical experience of targeted therapies that both approaches are less dichotomized than we previously hoped [30]. There are a number of significant side effects, mostly due to the fact that the targets are not specific for cancer cells.

The tyrosine kinase inhibitor imatinib leads to significant growth retardation in children receiving long-term CML treatment [98]. Second and third generation BCR/ABL tyrosine kinase inhibitors are associated with several vascular adverse effects like pulmonary hypertension and occlusive events [99]. CD19 CAR T-cell therapy is sometimes associated with severe side effects and toxicities such cytokine release syndrome associated and CNS endothelial cell activation-associated neurotoxicity, due leading to disruption of the blood-brain barrier [100]. Both can be life threatening and occasionally fatal. The permanent depletion of B cells by CD19 CAR T-cells leads to an increased risk of infections similar to that observed in primary treatment or salvage of patients with advanced ALL [101]. Autoimmune adverse events are common in the treatment with checkpoint inhibitors. They appear in 85% of melanoma patients treated with cytotoxic T-lymphocyte-associated protein 4 (CTLA-4) antibody ipilimumab [102] and even with a delay of several months after treatment [103].

Finally, since signaling in most cancers is not hard wired by oncogene addiction pathways and the Heisenberg principle applies to cancer heterogeneity and plasticity, targeted therapies may well prime for resistance: the targeted pathway can be bypassed by alternate rescue signaling [30].

Apart from these side effects and drawbacks, the financial toxicity potential of precision medicine for the health care system has also to be taken into account. The costs for the treatment of one pediatric neuroblastoma patient with dinutuximab beta amount to 173,000€ [104] depending on his body surface. The cost for CD19 CAR T-cell therapy tisagenlecleucel is 475,000$ exceeding the cost for conventional chemotherapy for B cell acute leukemia by almost 330,000$ [105]. A substantial part of this toxicity is a consequence of the regulatory fundamentalism in dealing with of advance therapy medicinal products (ATMPs).

## Conclusions

Current successful therapies in childhood cancer come at a high cost, e.g., secondary malignancies, developmental problems, cognitive decline, and early aging. Thus, development of precision medicine in pediatric oncology is an urgent medical need in public interest.

## Abbreviations

ABL: Abelson murine leukemia viral oncogene homolog 1
ADCC: Antibody-dependent cellular cytotoxicity
ALL: Acute lymphoblastic leukemia
BCG: Bacille Calmette-Guérin
BCL-2: B-cell leukemia 2
BCR: Breakpoint cluster region
BiTE: Bi-specific T-cell engagers
CAR: Chimeric antigen receptor
CDC: Complement dependent cytotoxicity
CHMI: Chondromodulin I
CTLA4: Cytotoxic T-lymphocate-associated antigen 4
DKFZ: German cancer research center
EBMT: European Society for Blood and Marrow Transplantation
ES: Ewing sarcoma
EZH2: Enhancer of zeste homolog 2
GFI1: Growth factor independent 1 trascriptional factor
GPOH: Society for Pediatric Oncology and Hematology
HLA: Human leukocyte antigen
ITP: Idiopathic thrombocytopenia
MATCH: Molecular analysis for therapy of cancer
MHC: Major histocompatibility complex
MYCN: **My**elo**c**ytomatosis oncogene of neuroblastoma
NIH: National Institute of Health
NK: Natural killer cell
Ph: Philadelphia
PTLD: Post transplant lymphoproliferative disease
SHH: Sonic hedgehog
SMO: Smoothened protein
TERT: Telomerase reverse transcriptase
tyr: Tyrosine
WNT: Wingless
