# Correction to: Precision medicine in pediatric oncology

**DOI:** 10.1186/s40348-022-00140-4

**Published:** 2022-03-10

**Authors:** Stefan E. G. Burdach, Mike-Andrew Westhoff, Maximilian Felix Steinhauser, Klaus-Michael Debatin

**Affiliations:** 1 Department of Pediatrics and Children’s Cancer Research Center (CCRC), Technische Universität München, Koelner Platz 1, 80804 Munich, Germany; 2 Department of Pediatrics and Adolescent Medicine, Ulm University Medical Center, Eythstr. 24, 89075 Ulm, Germany; 3 CCC München-Comprehensive Cancer Center and German Translational Cancer Research Consortium (DKTK), Partner Site Munich, Munich, Germany

**Correction to: Mol Cell Pediatr 5, 6 (2018)**

**https://doi.org/10.1186/s40348-018-0084-3**

Following publication of the original article [1], the author would like to correct the incorrect grant number found in the Funding statement.

Incorrect Funding statement:

“This work was supported by grants from Cura Placida Children’s Cancer Research Foundation and the Wilhelm-Sander Stiftung (2009.901.3). It is part of the Prospective Validation of Biomarkers in Ewing Sarcoma for Personalised Translational Medicine (PROVABES; 01KT1311), Funding Program of the Federal Ministry of Education and Research (BMBF), Germany.”

Correct Funding statement:

“This work was supported by grants from Cura Placida Children’s Cancer Research Foundation and the Wilhelm Sander-Stiftung (2015.028.1). It is part of the Prospective Validation of Biomarkers in Ewing Sarcoma for Personalised Translational Medicine (PROVABES; 01KT1311), Funding Program of the Federal Ministry of Education and Research (BMBF), Germany.

The original article has been corrected.

## References

[1] Burdach SEG, Westhoff MA, Steinhauser MF (2018). Precision medicine in pediatric oncology. Mol Cell Pediatr.

